# Temporal heterogeneity of microbial ecosystems and its formation mechanisms in Moutai-flavor Baijiu fermentation

**DOI:** 10.3389/fmicb.2026.1798174

**Published:** 2026-03-12

**Authors:** Zhiyu Zhu, Xiaobo Li, Tongbi Cui, Zongxiao Chen, Yong Liu, Xiao Chen, Yuan Tian, Yu Mu, Yuzhang Wu, Qing Ji, Sui Yan, Yanbo Cheng

**Affiliations:** 1 Kweichow Moutai Co. Ltd., Renhuai, Guizhou, China; 2 Moutai Institute, Renhuai, Guizhou, China

**Keywords:** cooling yard surface basicity, fermentation microbiome, high-throughput sequencing, microbial community adaptation, Moutai-flavor Baijiu, workshop age

## Abstract

The influence of workshop age on Moutai-flavor Baijiu fermentation is recognized, but the mechanisms driving microbial community shifts remain unclear. Understanding how the physical environment selects for specific microbiota is crucial for optimizing new workshops. Through 16S/ITS sequencing of pit-entry fermented grains, Daqu, air, and cooling yards in 5-, 10-, 20-, and 30-year-old workshops, *Lactobacillus* emerged as a key discriminant genus, increasing from 15.02% (5-year) to 35.59% (30-year). SourceTracker analysis revealed the cooling yard as the primary microbial source, contributing 54.2% on average to fermented grains. CO_2_-TPD analysis showed a 3.6-fold reduction in cooling yard surface basicity (from 0.11 to 0.03 mmol·g^−1^) over 30 years, resulting in high abundances (>10%) of alkalotolerant bacteria (e.g., *Alkalibacterium*, *Nesterenkonia*) and low *Lactobacillus* (2.17%). *Nesterenkonia* was also a biomarker in 5-year fermented grains. This confirms cooling yard surface basicity drives microecological differences, revealing how long-term production practices domesticate microbial communities and providing a theoretical basis for new workshop adaptation.

## 1 Introduction

Chinese Baijiu is a solid-state fermented distilled spirit carrying intangible cultural heritage, with a long brewing history and diverse aroma characteristics (Liu and Sun, 2018). In 2024, 989 large-scale Baijiu enterprises in China achieved a total output of 4.1 million kiloliters. Their sales revenue and profits reached 796.38 billion yuan and 250.87 billion yuan, respectively. These figures highlight the significant industrial and cultural value of the Chinese Baijiu industry. As a representative of Moutai-flavor Baijiu, Moutai originated in the Chishui River Basin, Guizhou Province (Supplementary Figure S1). The unique high-temperature, high-humidity climate, coupled with purple sandy shale soil (rich in iron, calcium, and other minerals), provides an irreplaceable microhabitat supporting the metabolic activities of brewing microorganisms. These factors constitute the core geographical elements that shape the regional flavor characteristics (Wang et al., 2015). Moutai-flavor Baijiu adopts an open solid-state dual fermentation process, in which microbe-environmental interactions drive rapid dynamic shifts in temperature, pH, and oxygen within the fermented grains. This selectively enriches key functional microbiota such as *Lactobacillus*, thermophilic *Bacillus*, and ester-producing yeasts, ultimately yielding the distinctive sensory profile of “pronounced sauce aroma and persistent empty-glass lingering fragrance” (Li et al., 2024; Xu et al., 2023).

The diversity and succession patterns of microbial communities in fermented grains directly determine fermentation quality, with their origins relying on two key processes. The first stage is steamed grains cooling and Daqu inoculation, in which the steamed grains are cooled to 30–35 °C in the cooling yard before blending with the Daqu. Moutai-flavor Daqu is produced using wheat as the primary material, which is crushed, mixed with Daqu starter and water, and then compacted into a turtle-back shape. It undergoes 40 days of fermentation followed by 6 months of storage to develop its saccharification, fermentation, and aroma-producing functions (Wang et al., 2017; Zhao et al., 2023). Daqu powder is rich in functional microorganisms including *Bacillus*, *Lactobacillus*, and *Aspergillus*, which not only degrade macromolecules (e.g., starch and proteins) in raw materials but also serve as critical contributors to the flavor profile of Baijiu (Yang et al., 2021). The second stage is stacking fermentation. After inoculation with Daqu starter, the steamed grains are formed into hemispherical stacks in the cooling yard. This open aerobic fermentation facilitates the enrichment of environmental microorganisms from air, surfaces of the cooling yard, and tools (Yang et al., 2023). The stacking fermentation stage plays a critical role in driving physicochemical processes, such as bioheat accumulation, enzymatic browning, and Maillard reactions. These processes are driven by the full harnessing of environmental microorganisms. Consequently, this phase not only shapes the microbial community structure of the fermented grains but also serves as the core process for generating Moutai-aroma flavor compounds (Wang, 2022). In these two processes, the prolonged contact among Daqu, cooling yard, air, and fermented grains establishes a stable microbial input network, which likely acts as the primary source of core functional microbiota for brewing. However, the specific contributions of these components remain to be further investigated.

In recent years, the soaring market demand for Moutai-flavor Baijiu has prompted the production of numerous new fermentation workshops. Studies demonstrate that prolonged workshop age and natural microbial succession in aged fermentation workshops promote stable symbiotic microorganism-environment relationships through metabolic coadaptation. In contrast, new fermentation workshops exhibit pronounced microbial competition and fragile community structures owing to the deficiency of micro-environmental acclimatization (Miao et al., 2023; Niu et al., 2022). Although some studies have compared the microbial compositional differences between new and old fermentation workshops, the underlying mechanisms driving these disparities remain controversial. Moreover, existing research has yet to systematically analyze the impact of workshop age on the microbial communities in fermented grains and identify the key driving factors.

Therefore, this study systematically compared the microbial diversity differences in pit-entry fermented grains across fermentation workshops with varying operational durations (5, 10, 20, and 30 years) via amplicon sequencing. The contribution rates of Daqu, cooling yard, and air to the microbial community structure of fermented grains were further analyzed. Furthermore, by analyzing the elemental composition and total surface alkalinity of the tri-composite cooling yard (comprising lime, purple-red clay, and coal slag), the impact mechanism of the physicochemical properties of the cooling yard on the microecological structure of fermented grains was revealed. These findings enhance our understanding of the temporal dynamics in Moutai-flavor Baijiu brewing microecology and provide theoretical support for the precise acclimatization of new fermentation workshops.

## 2 Materials and methods

### 2.1 Sample collection

Samples were collected from a Moutai-flavor Baijiu production company located in Moutai, China.

#### 2.1.1 Microbial detection samples

Daqu, cooling yard, air, and pit-entry fermented grains were collected from 25 workshops of different ages (5, 10, 20, and 30 years).

##### 2.1.1.1 Daqu samples

Daqu that had been stacked in the cooling yard for 1 day was collected, and the quartering method was used for sampling (*n* ≈ 100).

##### 2.1.1.2 Cooling yard samples

Microorganisms on the ground of the cooling yard were collected by wiping with sterile cotton balls (randomly 8 points), and all cotton balls were mixed as one sample (*n* ≈ 105).

##### 2.1.1.3 Air samples

Air samples were collected using a portable high-flow impaction air sampler with a matched sterile filter membrane and sterilized sampling head [flow rate: 28.3 L/min, filter membrane pore size: 0.22 μm (*n* ≈ 70)].

##### 2.1.1.4 Pit-entry fermented grains samples

Samples were collected from 8 random points from the surface to the center of the pile to be cellared, collected by the quartering method and placed in sealed bags (*n* ≈ 90).

Sampling was conducted from the 1st round to the 9th round of Moutai-flavor Baijiu production in Moutai Town.

#### 2.1.2 Traditional lime-clay composite soil material detection samples

Traditional lime-clay composite soil (TLCS) is a material for the workshop cooling yard of Moutai-flavor Baijiu production, which is prepared by mixing quicklime, purplish-red mud, and cinder. TLCS cooling yard samples were collected from 4 production teams, including a newly commissioned workshop with production history less than 1 year (sampling of three components: quicklime, purplish-red clay, and cinder) and a workshop with 30 years of production. All samples were immediately transported back to the laboratory for immediate analysis and research (within 24 h), and the samples retained in the laboratory were stored in a −20 °C freezer.

### 2.2 Microbiome DNA extraction, amplicon library preparation and sequencing data processing

Microbiome DNA was extracted via a magnetic bead-based protocol: samples were homogenized with grinding beads in Buffer ATL/PVP-10, centrifuged, and then purified automatically on a Kingfisher instrument using magnetic bead buffers containing Proteinase K and RNase A, with purified DNA eluted into centrifuge tubes. Subsequently, amplicon libraries were constructed by amplifying variable regions of bacterial 16S rDNA (V3/V4) and fungal ITS rDNA (ITS1/ITS2) using 2 × Phanta Max Master Mix and degenerate PCR primers; PCR products were purified with DNA magnetic beads and sequenced on the Illumina MiSeq platform to generate 2 × 250 bp paired-end reads. Raw sequencing data were filtered to remove low-quality, adapter-contaminated, ambiguous-base, and low-complexity reads (He et al., 2013), after which overlapping paired-end reads (minimum overlap length 15 bp, mismatch ratio ≤ 0.1) were merged with FLASH (v1.2.11) (Magoc and Salzberg, 2011); reads were then clustered into OTUs at 97% similarity using USEARCH (chimeras filtered by UCHIME), and taxonomically annotated via RDP Classifier (sequence identity threshold 0.6), with unannotated OTUs and those mismatched with the research background excluded.

### 2.3 Analysis of TLCS material and its components

(1) The elemental content of the bulk was analyzed by Malvern Panalytical Zetium X-ray fluorescence (XRF) spectrometer.

(2) CO_2_-temperature programmed desorption (CO_2_-TPD) tests were conducted with the sample being preheated at 300 °C for 1 h under He gas flow, and then cooled to 50 °C to allow CO_2_ gas adsorption for 30 min. After increasing the temperature to 800 °C at a heating rate of 10 °C/min, desorption was carried out.

### 2.4 Statistical analysis

The “vegan” package in R software (v 4.1.1; https://www.r-project.org/) was utilized to assess the *α*-diversity (including Chao1 and Shannon indices) and *β*-diversity of microbial communities, such as principal coordinate analysis (PCoA) and non-metric multidimensional scaling (NMDS) based on Bray-Curtis distance. The linear discriminant analysis effect size (LEfSe) was employed to identify the biomarker of microbial communities, conducted via an open-access pipeline with the thresholds of LDA > 3.0 and *p* < 0.05^1^.

## 3 Results

### 3.1 Effects of workshop age on microbial community structure of fermented grains

After undergoing steaming, cooling, Daqu starter addition, and stacking fermentation, the fermented grains of Moutai-flavor Baijiu that meet the pit-entry criteria are referred to as pit-entry fermented grains. Its microbial diversity represents a comprehensive manifestation of the entrapment, screening, and enrichment of microorganisms derived from the environment and Daqu by the fermented grains. High-throughput sequencing results showed that the bacterial populations in pit-entry fermented grains belonged to 20 phyla, among which 4 phyla had an average relative abundance (ARA) > 0.10% (Supplementary Figure S2a), namely Firmicutes (87.87%), Bacteroidetes (5.74%), Proteobacteria (5.58%), and Actinobacteria (0.39%). The ARA of Bacteroidetes in pit-entry fermented grains exhibited a numerical tendency for decrease as the workshop age increased. Compared with the 5-year-old workshop, the ARA in the 30-year-old workshop decreased from 10.54 to 2.26%, representing a 4.6-fold reduction. Studies have shown that Bacteroidetes prefer neutral to alkaline environments, suggesting that there may be differences in the pH of the brewing environment among workshops of different ages (Hellequin et al., 2023).

A total of 9 phyla were detected in the fungal communities of pit-entry fermented grains, with 4 phyla showing an ARA > 0.10% (Supplementary Figure S2b): Ascomycota (88.55%), Apicomplexa (8.66%), Mucoromycota (2.31%), and Basidiomycota (0.48%). No age-related gradient differences were detected across workshops.

**Figure 1:**
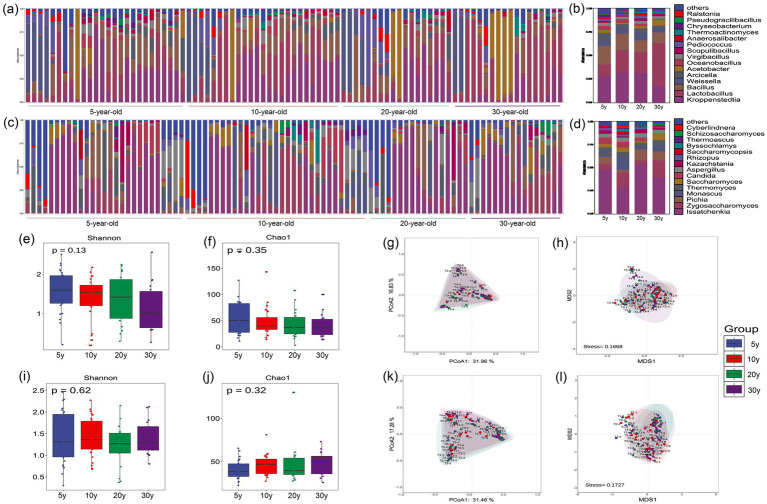
Microbial community structure and diversity analysis of pit-entry fermented grains across different workshop age groups. **(a)** ARA distribution of the top 15 dominant bacterial genera across all samples. **(b)** Comparative analysis of ARA among different workshop age groups. **(c)** ARA distribution of the top 15 dominant fungal genera across all samples. **(d)** Comparative analysis of ARA among different workshop age groups. **(e)** Bacterial α-diversity (Shannon index). **(f)** Bacterial α-diversity (Chao1 index). **(g)** Bacterial β-diversity (PCoA). **(h)** Bacterial β-diversity (NMDS). **(i)** Fungal α-diversity (Shannon index). **(j)** Fungal α-diversity (Chao1 index). **(k)** Fungal β-diversity (PCoA). **(l)** Fungal β-diversity (NMDS). Statistical Analysis: Differences between groups were analyzed using one-way ANOVA. Error bars represent the standard deviation (SD) of biological replicates.

A total of 1,020 bacterial genera were detected in the pit-entry fermented grains, with *Kroppenstedtia*, *Lactobacillus*, and *Bacillus* identified as the dominant genera (Figures 1a,b). *Kroppenstedtia* represents a pivotal dominant bacterial genus in Moutai-flavor Baijiu, characterized by three core functional traits: thermotolerance, flavor metabolism, and ecological niche competitiveness. Its robust enzyme-producing capacity and antibacterial mechanisms play significant roles in maintaining the acid–base balance of the fermentation system and indirectly promoting the accumulation of high-temperature flavor compounds (Chen et al., 2022). *Lactobacillus* serves as the dominant bacterial group throughout both the stacking fermentation and pit fermentation stages of Moutai-flavor Baijiu production. It makes significant contributions to the synthesis of lactic acid-dominated flavor precursor substances, while its ARA is significantly higher in aged fermentation pits compared to new ones-establishing it as a signature microorganism for the pit fermentation process. Similarly, *Lactobacillus* serves as a core bacterial genus in fermented grains from mature workshops producing Luzhou-flavor Baijiu. Its metabolite, lactic acid, exerts profound environmental acclimation effects on the brewing ecosystem, inhibiting the growth of miscellaneous bacteria by creating a stable low-pH environment and promoting the accumulation of Baijiu flavor substances through cross-niche metabolic cooperation (Hao et al., 2021; Li et al., 2022). *Bacillus* possesses efficient carbon metabolic capacities, with its metabolites containing abundant core flavor compounds of Moutai-flavor Baijiu, such as tetramethylpyrazine. It can synergistically optimize the alcohol content of the fermented Baijiu with yeast while inhibiting the production of fusel alcohols, thus establishing it as a prevalent dominant functional bacterial genus in high-temperature Daqu used for Moutai-flavor Baijiu production (Wang et al., 2023).

Bacterial α-diversity analysis (Figures 1e,f) revealed no significant difference (*p* > 0.05). However, the median diversity indices exhibited a numerical tendency for decrease as workshop age increased, with the 5-year-old group showing the highest values (Shannon 1.58, Chao1 50.62) and the 30-year-old group presenting the lowest values (Shannon 1.15, Chao1 38.5). The results indicated that new workshops showed inadequate accumulation of microorganisms and metabolites, with its microbial community still in the early stage of ecological succession. This successional status renders the environment more susceptible to exogenous microbial colonization, thereby leading to higher α-diversity in the pit-entry fermented grains (Li et al., 2022). As workshop age increases, the brewing ecosystem undergoes gradual acclimation. The dominant functional brewing microorganisms gradually gained dominance in community assembly while inhibiting the proliferation of non-functional contaminant microorganisms, resulting in a marked reduction in bacterial diversity index in the pit-entry fermented grains after 30 years of production. Bacterial β-diversity analysis (Figures 1g,h) showed substantial overlap among confidence ellipses of the four sample groups overall, whereas the 30-year-old group exhibited distinct clustering in both ordination plots. This clustering pattern suggests higher inter-sample consistency in bacterial community composition and a more stable core microbiota structure. In contrast, the 5-year-old and 10-year-old groups showed greater dispersion with increased community heterogeneity, suggesting that microbial communities in newer workshops are more prone to exogenous colonization and exhibit larger compositional fluctuations.

A total of 701 fungal genera were detected, with *Issatchenkia*, *Zygosaccharomyces*, and *Pichia* identified as the dominant genera (Figures 1c,d). *Issatchenkia*, is a well-recognized core yeast genus in Moutai-flavor Baijiu stacking fermentation, exhibits robust tolerance to high-temperature and high-ethanol stress environments. It possesses strong synergistic metabolic capacities, including esterification and organic acid homeostasis regulation, which play critical roles in facilitating the biosynthesis of flavor compounds and maintaining acid–base homeostasis within the fermentation ecosystem (Shen et al., 2022). *Zygosaccharomyces* is a prevalent fungal genus in the high-temperature stacking fermentation stage of Moutai-flavor Baijiu, characterized by robust ethanol-producing and esterification capabilities. It has evolved intricate heat stress response pathways and displays remarkable lactic acid stress tolerance during the stacking fermentation stage (Gao et al., 2022). *Pichia* demonstrates robust lactic acid tolerance and thermostability, with the capacity to metabolize key flavor-active compounds (including 2-phenylethanol and isoamyl alcohol) and various ester precursors under high lactic acid stress. This metabolic activity alleviates excessive lactic acid accumulation during the “high-temperature stacking” phase of Moutai-flavor Baijiu production while preserving microbial community stability within the brewing ecosystem (Deng et al., 2020).

Fungal *α*-diversity analysis (Figures 1i,j) revealed no statistically significant differences among groups (*p* > 0.05). The median Shannon index across all four sample groups ranged from 1.2 to 1.5, while the median Chao1 index fluctuated between 40 and 60, indicating that neither species richness nor community evenness exhibited significant variation with workshop age. Fungal β-diversity analysis (Figures 1k,l) demonstrated substantial overlap in the confidence ellipses of the four groups, with no clear inter-sample clustering pattern observed. This suggests high similarity in fungal community composition across different workshop age groups.

All these dominant microorganisms identified in pit-entry fermented grains represent core functional taxa in the brewing microbiome, playing pivotal roles in sustaining fermentation system homeostasis and ensuring the quality consistency of base Baijiu.

### 3.2 Temporal heterogeneous microorganisms in production workshops of different ages

**Figure 2:**
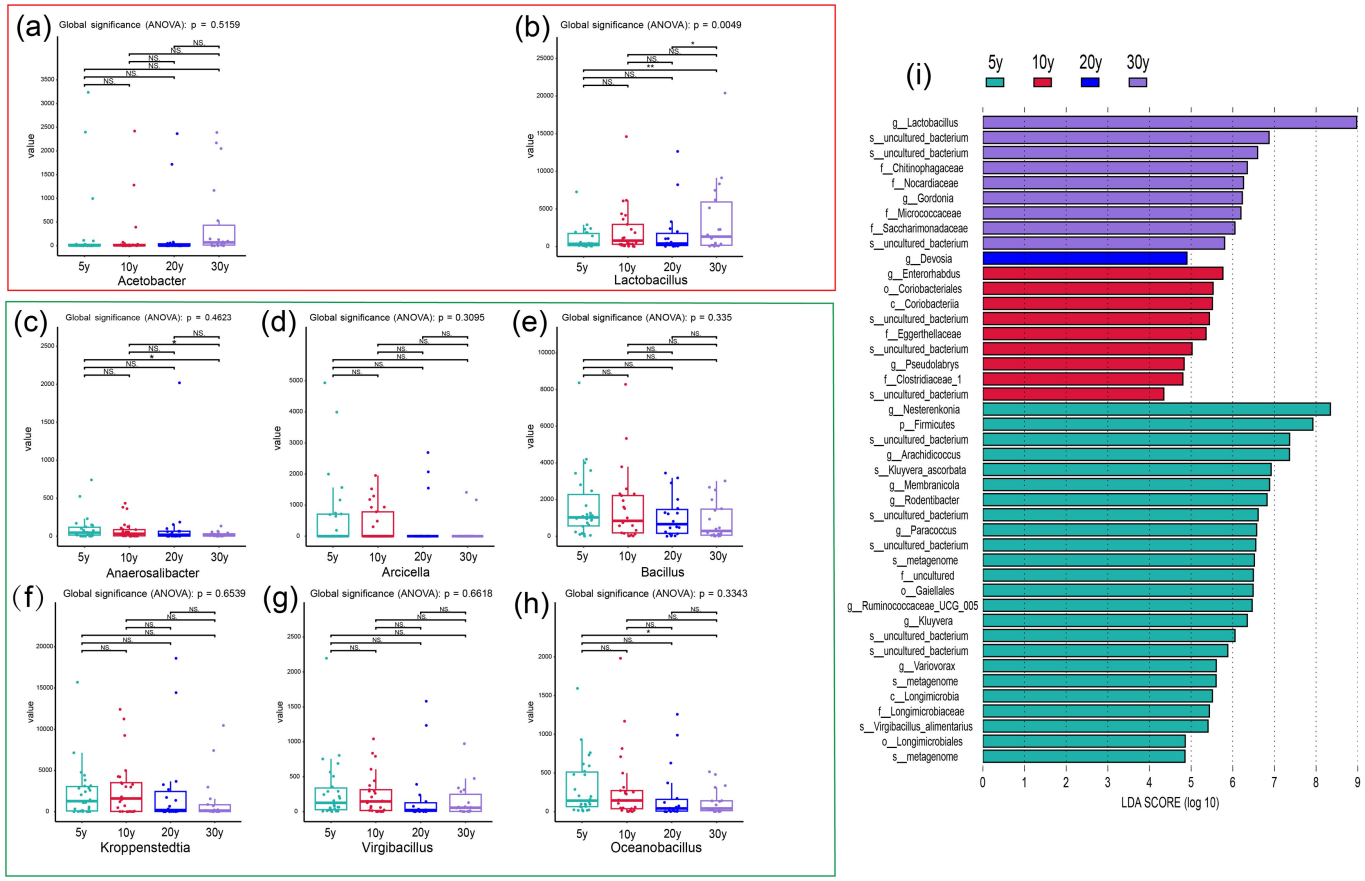
Temporal heterogeneity analysis of bacterial ARA at the genus level in pit-entry fermented grains. **(a,b)** Dominant bacterial genera showing increasing ARA with increasing workshop age. **(c–h)** Dominant bacterial genera showing decreasing ARA with increasing workshop age. Brackets indicate statistical comparisons between groups. ns, not significant; *p < 0.05. **(i)** LEfSe-identified taxonomic biomarkers of bacterial communities in pit-entry fermented grains across different workshop age groups (LDA score > 4.0, *p* < 0.05).

Furthermore, Taxonomic analysis at the dominant bacterial genus level demonstrated that *Acetobacter*, *Lactobacillus*, *Anaerosalibacter*, *Arcicella*, *Bacillus*, *Kroppenstedtia*, *Virgibacillus* and *Oceanobacillus* exhibited significant abundance variations associated with workshop age (Figures 2a–h). Among them, the ARA of *Acetobacter* and *Lactobacillus* exhibited a numerical tendency for increase with increasing workshop age. In the 30-year-old group, the ARA of *Lactobacillus* was relatively high (45.75%), which was 33.68, 22.07, and 23.99% higher than that in the 5-year-old, 10-year-old, and 20-year-old groups, respectively. Significant differences were observed between the 30-year-old group and both the 5-year-old and 20-year-old groups, with an extremely significant difference detected between the 30-year-old and 5-year-old groups; furthermore, extremely significant differences across all four sample groups were identified (*p* = 0.0049) (Figure 2b). In the 30-year-old group, the ARA of *Acetobacter* was relatively high (6.27%), which was 3.01, 4.30, and 3.42% higher than those in the 5-year-old, 10-year-old, and 20-year-old groups, respectively, however, these differences were not statistically significant (Figure 2a). Both genera exhibit dual acidophilic and acidogenic traits, and their growth, reproduction, and metabolic processes are adapted to acidic environmental conditions (typically pH 3.0–5.5) (Zahan et al., 2015).

However, the ARA of *Anaerosalibacter*, *Arcicella*, *Bacillus*, *Kroppenstedtia*, *Virgibacillus*, and *Oceanobacillus* exhibited a numerical tendency for decrease with increasing workshop age. In the 5-year-old group, the ARA of *Kroppenstedtia* was 22.52%, which was 12.30% higher than that in the 30-year-old group (Figure 2f). The ARA of *Bacillus* in the 5-year-old group was 18.27%, which was 9.51% higher than that in the 30-year-old group (Figure 2e). The ARA of *Arcicella* in the 5-year-old group was 4.95%, which was 4.35% higher than that in the 30-year-old group (Figure 2d). The ARA of *Oceanobacillus* in the 5-year-old group was 3.67%, which was 2.44% higher than that in the 30-year-old group, with a statistically significant difference (Figure 2h). The ARA of *Virgibacillus* in the 5-year-old group was 2.98%, which was 1.61% higher than that in the 30-year-old group (Figure 2g). The ARA of *Anaerosalibacter* in the 5-year-old group was 1.15%, which was 0.61% higher than that in the 30-year-old group, with a significant difference (Figure 2c). Microorganisms showing distinct ARA advantages in the 5-year-old group exhibit prominent halotolerant and thermotolerant traits. Regarding growth environment adaptation, the growth, reproduction, and metabolic activities of these microorganisms are better adapted to neutral to alkaline environmental conditions (Rezgui et al., 2012; Sheu et al., 2010; Heyndrickx et al., 1998; Heyrman and De Vos, 2015).

In contrast, the overall composition of fungal communities remained relatively stable, with only *Saccharomyces* and *Zygosaccharomyces* exhibiting significant abundance variations associated with workshop age. This stability likely stems from the intrinsically high stress resistance and environmental tolerance of fungi against fermentation-related physicochemical fluctuations (Zhang et al., 2022). Unlike bacteria, which often rely on rapid taxonomic shifts to adapt to changing conditions (e.g., the dominance shift of *Lactobacillus*), fungi appear to employ a more generalized survival strategy, maintaining a consistent community structure while selectively enriching specific functional taxa. This suggests a different ecological adaptation model where fungi maintain resilience through physiological tolerance rather than community restructuring. Specifically, the ARA of *Saccharomyces* in the 30-year-old group was 7.44%, which was 3.77% higher than that in the 5-year-old group (Supplementary Figure S3j). Similarly, the ARA of *Zygosaccharomyces* in the 30-year-old group was 17.93%, a statistically significant increase of 12.16% compared to the 5-year-old group (Supplementary Figure S3o). Consistent with the acid-tolerant *Lactobacillus*, both of these fungi are well-adapted to slightly acidic environments (Parapouli et al., 2020). This shared ecological niche explains their parallel enrichment in the 30-year-old group, suggesting that both bacterial and fungal communities are ultimately governed by the same selective pressure of acidification during long-term fermentation.

Furthermore, LEfSe analysis was performed to characterize group-specific bacterial taxonomic structures and identify genus-level taxonomic biomarkers across the four workshop age groups. As shown in Figure 2i, a total of 43 taxonomic biomarkers were identified based on LDA scores. Notably, *Lactobacillus* in the 30-year-old group exhibited an extremely high LDA score (LDA = 8.97, *p* = 0.04), representing the core differential taxon for this group. Distinct microbial signatures were also observed in the 5-year-old and 10-year-old groups; however, only one biomarker was identified in the 20-year-old group, indicating weak community specificity. *Arachidicoccus* and *Nesterenkonia* serve as the key signature genera in the 5-year-old group. They are rare microbial genera specifically found in Moutai-flavor Baijiu production from new workshops and thrive in neutral to weakly alkaline environments (Lee et al., 2020; An et al., 2025). *Enterorhabdus* and *Pseudolabrys* are the dominant signature genera in the 10-year-old group; both adapt to neutral to slightly acidic environments and are also rare microbial genera in this specific production context (Clavel et al., 2009; Wei et al., 2022).

These results indicate that as workshop age increases, the internal microenvironment of the brewing system undergoes predictable directional changes, leading to more pronounced enrichment of acidophilic taxa. In contrast, alkaliphilic or neutrophilic taxa dominate and form distinct enrichment patterns in younger workshops. Collectively, the significant inter-group differences, the exceptionally high LDA score of *Lactobacillus* in the 30-year-old group, and its dominant abundance in pit-entry fermented grains demonstrate that *Lactobacillus* is the core functional genus shaping the microbiota variations across different workshop ages in Moutai-flavor Baijiu production.

### 3.3 Analysis of *Lactobacillus* in pit-entry fermented grains

The microbial communities in Moutai-flavor Baijiu fermented grains is primarily derived from Daqu and environmental sources, with the environmental sources including the cooling yard, air, production tools, and workers (Lu et al., 2023; Huang et al., 2024). Meanwhile, considering that the cooling yard and air exhibit the longest contact duration with fermented grains, they potentially harbor more intensive microbial interactions with the grains. Therefore, this study further investigated variations in microbial community composition across Daqu, air, and cooling yard samples from different workshop age groups, while quantifying the respective contribution of each source to *Lactobacillus* colonization in pit-entry fermented grains.

#### 3.3.1 Analysis of microbial composition in Daqu

**Figure 3:**
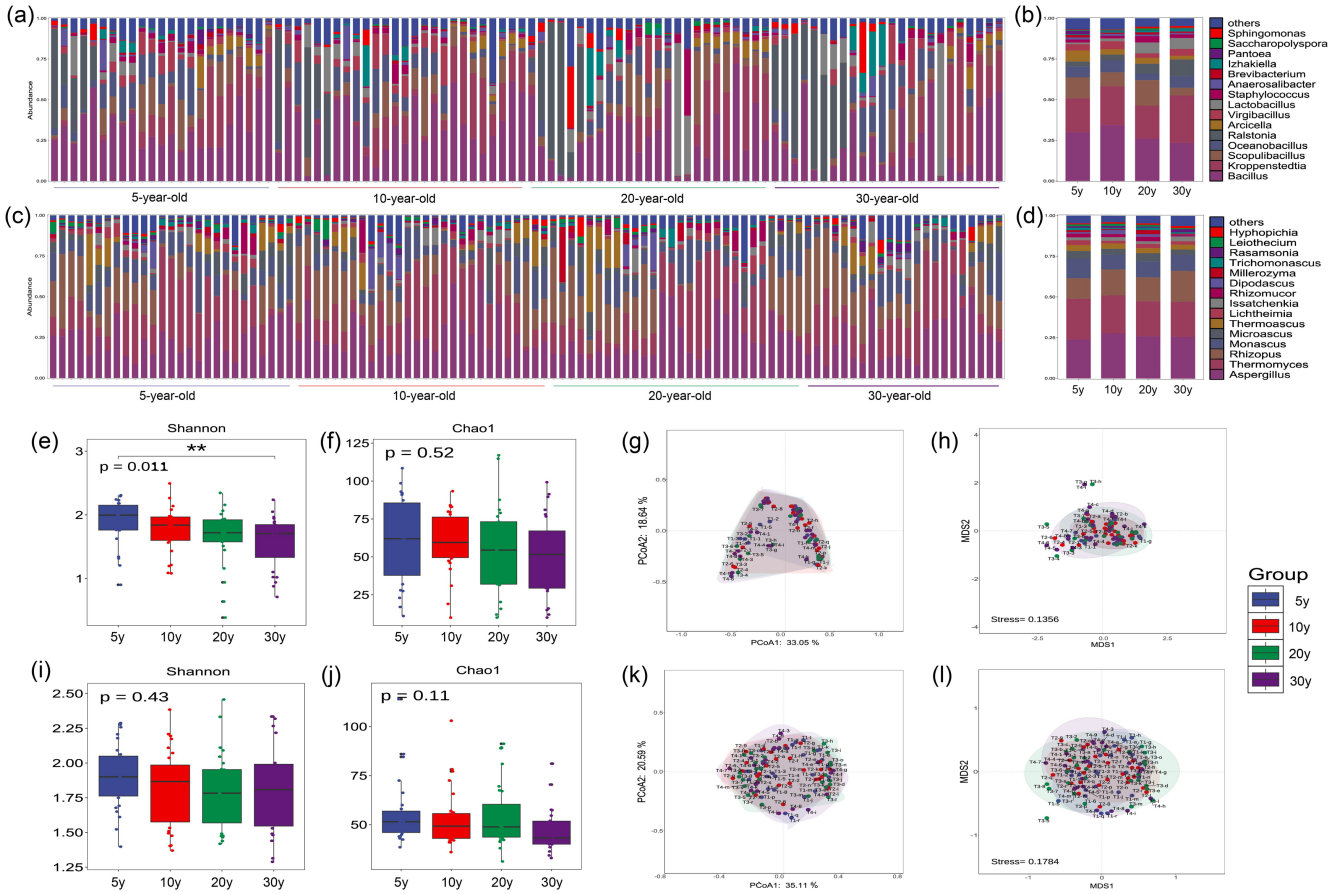
Microbial community structure and diversity analysis of Daqu from different workshop age groups. **(a)** ARA distribution of the top 15 dominant bacterial genera across all samples. **(b)** Comparative analysis of ARA among different workshop age groups. **(c)** ARA distribution of the top 15 dominant fungal genera across all samples. **(d)** Comparative analysis of ARA among different workshop age groups. **(e)** Bacterial α-diversity (Shannon index). **(f)** Bacterial α-diversity (Chao1 index). **(g)** Bacterial β-diversity (PCoA). **(h)** Bacterial β-diversity (NMDS). **(i)** Fungal α-diversity (Shannon index). **(j)** Fungal α-diversity (Chao1 index). **(k)** Fungal β-diversity (PCoA). **(l)** Fungal β-diversity (NMDS). Statistical Analysis: Differences between groups were analyzed using one-way ANOVA. Error bars represent the standard deviation (SD) of biological replicates.

A total of 482 bacterial genera were detected in Moutai-flavor Daqu, with *Bacillus*, *Kroppenstedtia*, and *Scopulibacillus* identified as the dominant genera (Figures 3a,b). A total of 224 fungal genera were identified, with *Aspergillus*, *Thermomyces*, and *Rhizopus* identified as the dominant genera (Figures 3c,d). These microorganisms represent core functional taxa in Moutai-flavor Daqu, exhibiting robust enzyme-producing capacities. Through carbohydrate metabolism, they play a crucial role in the synthesizing of important enzyme systems such as amylase and protease in Daqu, as well as the metabolism of diverse flavor precursor compounds (Zhu et al., 2023). The results of bacterial *α*-diversity analysis (Figures 3e,f) revealed a statistically significant difference in the Shannon index across the four workshop age groups (*p* = 0.011), with the median diversity indices decreasing as the workshop age increased. The 5-year-old group exhibited the highest diversity (Shannon = 2.00, Chao1 = 62.73), whereas the 30-year-old group had the lowest (Shannon = 1.73, Chao1 = 51.58). However, analysis of temporal variations of the dominant bacterial genera in Daqu (Supplementary Figure S4), revealed no dominant bacterial genera with significant age-related trends, and no statistically significant inter-group differences were observed (*p* > 0.05). Combined with the results of bacterial β-diversity analysis of Daqu (Figures 3g,h), the confidence ellipses among groups showed a high degree of overlap, indicating high overall similarity in bacterial communities among different Daqu samples, with potential variations in low-abundance bacterial taxa. Meanwhile, no statistically significant difference in *Lactobacillus* ARA was observed across Daqu samples (*p* = 0.45), suggesting that Daqu may not be the primary driver of *Lactobacillus* abundance differences in pit-entry fermented grains. The results of fungal *α*-diversity analysis (Figures 3i,j) showed that the median Chao1 index decreased with increasing workshop age, with no statistically significant differences detected among groups. Together with the relatively stable proportions of dominant fungal genera, absence of significant age-related trends, and high overlap of confidence ellipses across groups in the fungal *β*-diversity analysis of Daqu (Figures 3k,l), indicating high overall similarity in fungal communities among different Daqu samples.

#### 3.3.2 Analysis of airborne microbial composition

**Figure 4:**
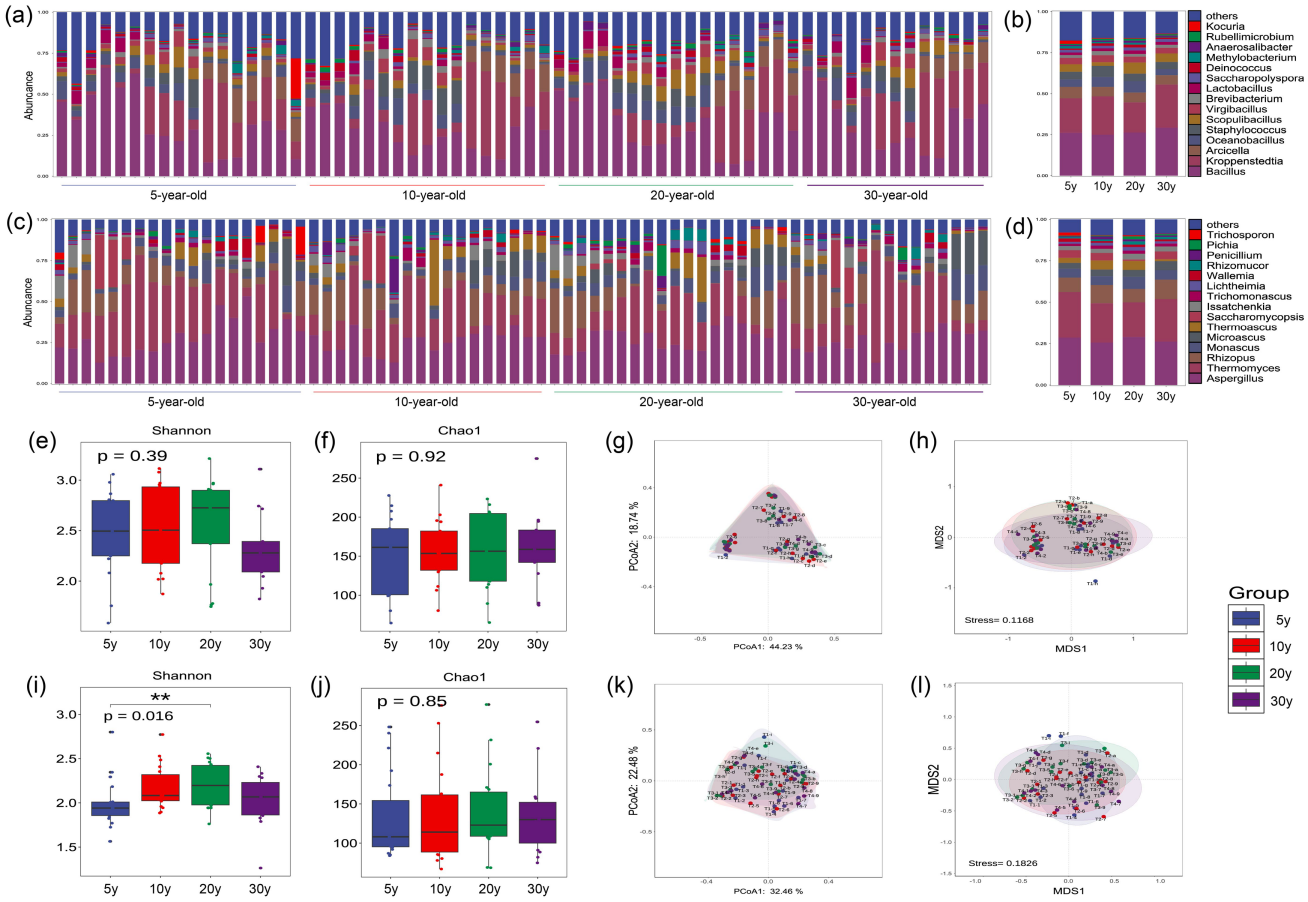
Analysis of airborne microbial community structure and diversity in workshops with different workshop age. **(a)** ARA distribution of the top 15 dominant bacterial genera across all samples. **(b)** Comparative analysis of ARA among different workshop age groups. **(c)** ARA distribution of the top 15 dominant fungal genera across all samples. **(d)** Comparative analysis of ARA among different workshop age groups. **(e)** Bacterial α-diversity (Shannon index). **(f)** Bacterial α-diversity (Chao1 index). **(g)** Bacterial β-diversity (PCoA). **(h)** Bacterial β-diversity (NMDS). **(i)** Fungal α-diversity (Shannon index). **(j)** Fungal α-diversity (Chao1 index). **(k)** Fungal β-diversity (PCoA). **(l)** Fungal β-diversity (NMDS). Statistical Analysis: Differences between groups were analyzed using one-way ANOVA. Error bars represent the standard deviation (SD) of biological replicates.

A total of 1,063 bacterial genera were detected in the workshop air, with *Bacillus*, *Kroppenstedtia*, and *Arcicella* predominating (Figures 4a,b). Among the 684 fungal genera identified, *Aspergillus*, *Thermomyces*, and *Rhizopus* were the most abundant (Figures 4c,d). The composition of these dominant taxa showed high similarity to that of Moutai-flavor Daqu, likely due to the environmental acclimation effect exerted by Daqu powder on the airborne microbial communities. While *Pseudomonas*, *Acinetobacter*, and other non-fermentative microorganisms are reportedly dominant in the air outside Moutai-flavor Baijiu workshops (Zhang, 2021), extensive dispersion of steam, Daqu powder, and other fermentation-related aerosols during internal operations significantly alters the intra workshop microbiota. This creates a pronounced divergence from the external environment. Bacterial α-diversity analysis (Figures 4e,f) revealed a relatively higher median Shannon index in the 20-year-old group (2.73) compared to the 30-year-old group (2.38); however, no statistically significant differences were observed overall, and the Chao1 index remained consistent across groups. Similarly, the median fungal Shannon index was significantly higher in the 20-year-old group (2.20) than in the 5-year-old group, although no clear gradient pattern was discernible (Figures 4i,j). Furthermore, PCoA and NMDS results (Figures 4g,h,k,l) indicated high overlap of confidence ellipses for both bacterial and fungal β-diversity across all groups. Notably, the ARA of *Lactobacillus* in the air remained consistently low (~2%) across different workshop age groups (Figure 4b) with no significant variation. This suggests that air may not be the primary driver of *Lactobacillus* abundance differences in pit-entry fermented grains.

#### 3.3.3 Analysis of microbial composition in cooling yard

**Figure 5:**
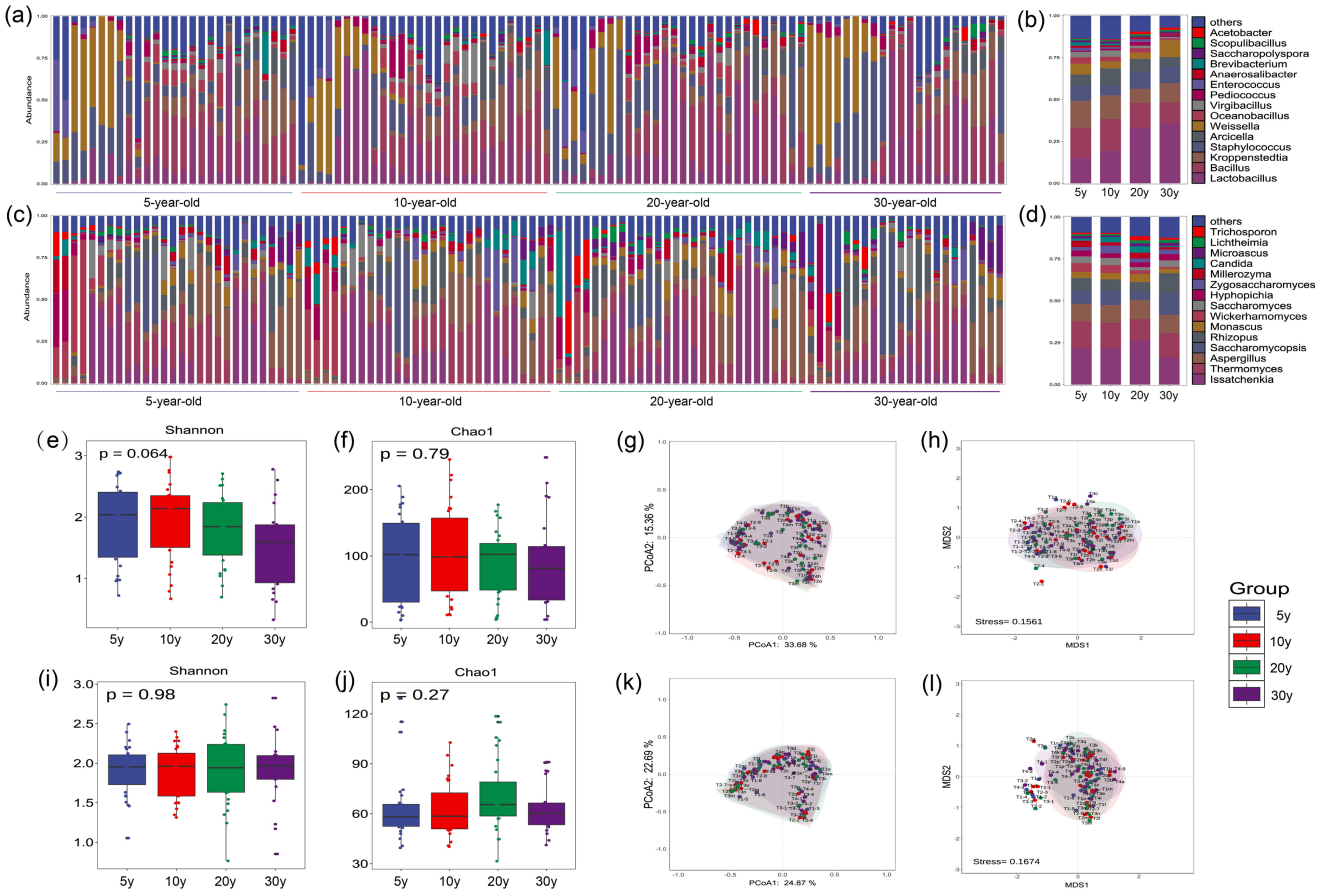
Analysis of microbial community structure and diversity in cooling yard from different workshop age groups. **(a)** ARA distribution of the top 15 dominant bacterial genera across all samples. **(b)** Comparative analysis of ARA among different workshop age groups. **(c)** ARA distribution of the top 15 dominant fungal genera across all samples. **(d)** Comparative analysis of ARA among different workshop age groups. **(e)** Bacterial α-diversity (Shannon index). **(f)** Bacterial α-diversity (Chao1 index). **(g)** Bacterial β-diversity (PCoA). **(h)** Bacterial β-diversity (NMDS). **(i)** Fungal α-diversity (Shannon index). **(j)** Fungal α-diversity (Chao1 index). **(k)** Fungal β-diversity (PCoA). **(l)** Fungal β-diversity (NMDS). Statistical Analysis: Differences between groups were analyzed using one-way ANOVA. Error bars represent the standard deviation (SD) of biological replicates.

A total of 639 bacterial genera were detected in the in Moutai-flavor Baijiu workshop cooling yard samples, with *Lactobacillus*, *Bacillus*, and *Kroppenstedtia* identified as the dominant genera (Figures 5a,b). A total of 688 fungal genera were identified, with *Issatchenkia*, *Aspergillus*, and *Thermomyces* identified as the dominant genera (Figures 5c,d). These microorganisms represent core functional taxa in Moutai-flavor Baijiu workshop cooling yard, possessing acidogenic, enzyme-producing, and flavor-enhancing functions, which play critical roles in maintaining the stability of the fermentation process (Zhu et al., 2024). Fungal communities in the cooling yard exhibited overall stability with no statistically significant differences among samples (Figures 5i–l), whereas bacterial communities showed notable variations (Figures 5e–h). In terms of bacterial α-diversity, the median Shannon index was relatively higher in the 10-year-old group (2.23) and lower in the 30-year-old group (1.57) (Figure 5e). Within the dominant bacterial taxa, the ARA of *Lactobacillus* exhibited a numerical tendency for increase with the increase of workshop age (15.02, 19.54, 32.22, and 35.59%) (Supplementary Figure S5e), while the ARA of *Oceanobacillus* exhibited a numerical tendency for decrease (3.74, 2.74, 2.66, and 1.13%) (Supplementary Figure S5j). Overall, the surface microbial community structure of cooling yards in different workshop age groups showed high similarity to that of pit-entry fermented grain microbiota. The high-temperature stacking fermentation process of Moutai-flavor Baijiu is also known as “secondary fermentation.” Throughout this process, the temperature of the fermented grains rises progressively. Concurrently, the accumulation of organic acids drives a gradual decline in pH, significantly altering the cooling yard environment (Hao et al., 2021). Cooling yards in older workshop age groups, after years of acclimatization through high-temperature stacking fermentation, may have significant changes in surface physicochemical properties and microbial community structure.

#### 3.3.4 SourceTracker-based traceability analysis of *Lactobacillus*

**Figure 6:**
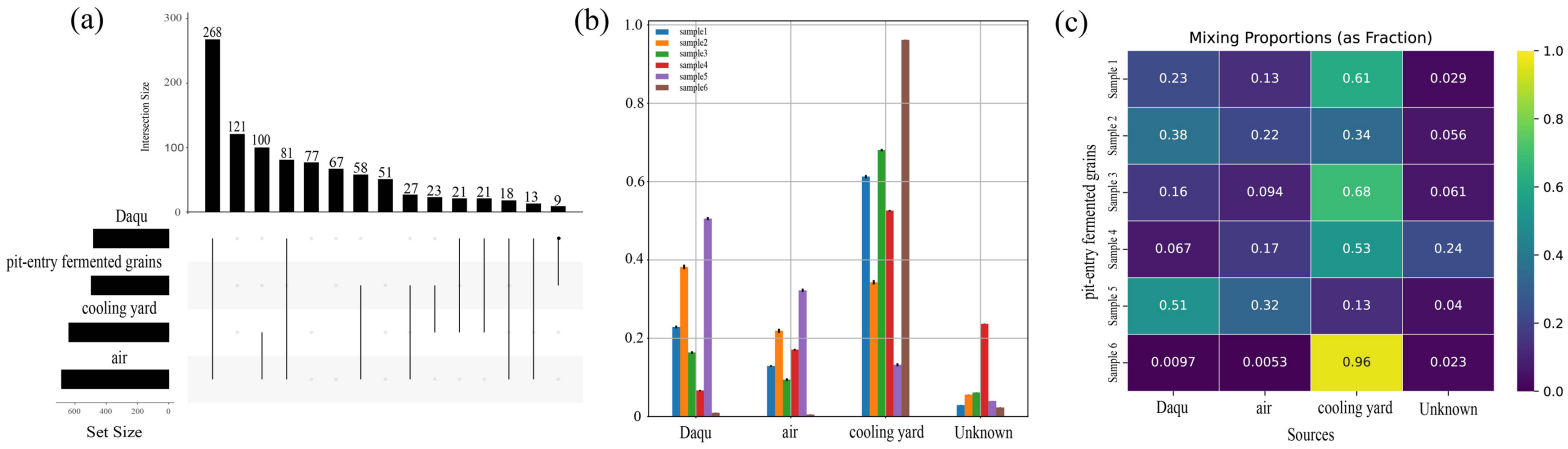
Traceability analysis of *Lactobacillus* in different key sources. **(a)** Upset plot analysis of bacterial composition across different key sources. **(b,c)** SourceTracker analysis plots of different key sources.

To identify the key sources (Daqu, air, and cooling yard) influencing the bacterial community of pit-entry fermented grains, microbial source tracking was performed using SourceTracker. As depicted in Figures 6b,c, the cooling yard was the predominant contributor to the bacterial community, accounting for up to 96% (mean 54.2%), followed by Daqu (maximum 51%, mean 22.6%) and air (maximum 32%, mean 15.7%), with unknown sources comprising a maximum of 24% (mean 10.9%). However, as a Bayesian model, SourceTracker’s inference is limited by source pool completeness. Thus, the 10.9% unknown fraction suggests potential contributions from un-sampled reservoirs (Knights et al., 2011). Furthermore, the cooling yard exhibited the highest community similarity to fermented grains (74.6%), surpassing both Daqu (62.7%) and air (73.8%) (Figure 6a). These results collectively indicate that the cooling yard governs the variations in *Lactobacillus* abundance. Notably, the abundance of *Lactobacillus* in cooling yards from 30-year-old workshops was 20.57% higher than that in 5-year-old workshops (Supplementary Figure S5e). This initial disparity was subsequently amplified during stacking fermentation, ultimately resulting in a 33.68% higher abundance of *Lactobacillus* in pit-entry fermented grains from the 30-year-old workshops compared to the 5-year-old workshops (Figure 2b). Given its dominant contribution among the identified sources and highest compositional similarity, we propose that, within the context of the sampled environments, the cooling yard plays a central role in shaping *Lactobacillus* abundance in fermented grains and may serve as the primary traceable source of *Lactobacillus* inoculum. This finding warrants further investigation into its physicochemical properties and microbiome to elucidate the underlying mechanisms.

### 3.4 Interaction between the physicochemical properties of the cooling yard and its microbial communities

The cooling yard serves as a critical site for the spreading and cooling steamed grains, as well as the enrichment of environmental microorganisms. Owing to long-term exposure to the external environment, the microbial community attached to its surface is significantly influenced by production operations, air, and personnel activities. The cooling yard is constructed with TLCS, a material prepared by mixing quicklime, purplish-red mud, and cinder in specific proportions. This material exhibits wear resistance and water absorbency, and is rich in inorganic elements. Previous studies have demonstrated that the material composition of the cooling yard exerts a significant regulatory effect on the diversity and functional profiles of surface-attached microorganisms, which is critical for the production of Moutai-flavor Baijiu and the formation of characteristic flavor compounds (Wang et al., 2021).

An XRF analyzer was employed to perform qualitative and quantitative analyses of the elemental composition of cooling yard and its individual components (quicklime, purplish-red mud, and cinder) (Table 1 and Supplementary Figure S6). The results indicated that the cooling yard primarily contains elements O, Si, Ca, Al, S, Fe, and Mg. Among these elements, Ca and Mg are predominantly derived from quicklime, Si, Al, and Fe are contributed by purplish-red mud and cinder, and S is mainly derived from cinder.

**Table 1:** Elemental composition and relative content of triad soil based on XRF (%).

Sample	Si	O	Ca	Al	S	Fe	Mg	K	Ti
TLCS	16.33	49.05	14.43	7.70	3.55	3.45	2.48	1.56	0.68
Quicklime	0.56	56.42	37.38	0.07	0.27	0.04	5.19	0.01	-
Purplish-red mud	27.66	55.16	0.09	10.50	0.02	3.17	0.77	1.84	0.44
Cinder	17.12	55.27	2.25	13.59	3.30	4.45	0.37	1.52	0.83

**Figure 7:**
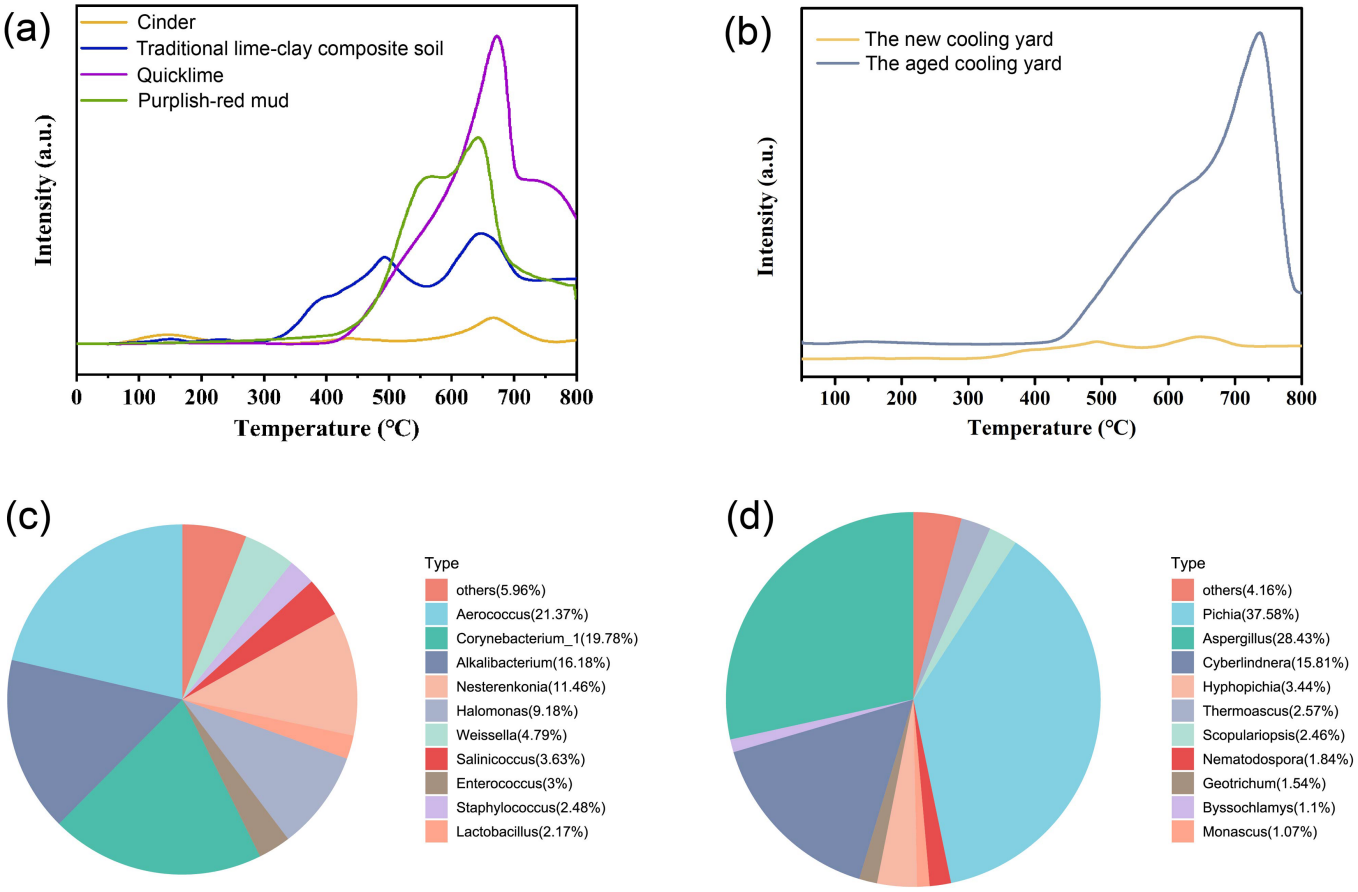
Compositions, physicochemical properties and surface microbial community structure of the cooling yard. **(a)** CO_2_-TPD analysis of cooling yard and its components. **(b)** CO_2_-TPD analysis of freshly prepared cooling yard and 30-year-old used cooling yard. **(c)** Top 10 bacterial taxa with the highest ARA. **(d)** Top 10 fungal taxa with the highest ARA.

Furthermore, CO_2_-TPD analysis was performed to characterize the surface basicity of the samples (Figure 7a). All samples exhibited strong basic sites, with the basic strength in the increasing order: cinder (0.03 mmol·g^−1^) < triad soil (0.11 mmol·g^−1^) < purplish-red mud (0.25 mmol·g^−1^) < quicklime (0.30 mmol·g^−1^). Given that both quicklime and purplish-red mud in TLCS are strongly alkaline, and the abundant Ca^2+^ in quicklime provides additional active sites for alkaline sites, the total alkali content of the TLCS cooling yard is significantly elevated (Tu et al., 2024). To investigate the variations in surface basicity of TLCS between fresh and aged cooling yards, this study compared the surface basicity of freshly prepared TLCS cooling yard (without prior involvement in production operations) and a 30-year-old in-service TLCS cooling yard (Figure 7b). A significant difference in the total surface alkali content between the new and 30-year-old commissioned TLCS cooling yard was observed. After 30 years of production, the total surface alkali content of the TLCS cooling yard decreased from 0.11 mmol·g^−1^ to 0.03 mmol·g^−1^. To further elucidate the mechanism behind this reduction, we analyzed the elemental composition of the TLCS samples. As shown in Supplementary Table S1, while the bulk Si/Al ratio remains relatively stable (fluctuating around 2.4), the Ca content exhibits a significant decreasing trend, dropping from an average of 76.94% (5-year-old) to 33.46% (30-year-old). The stability of the Si/Al ratio suggests a consistent framework structure (often associated with zeolitic or aluminosilicate phases) that acts as the backbone of the material (Corma, 1995). Consequently, the substantial depletion of Ca directly corresponds to the observed decline in surface alkalinity. This indicates that while the framework remains intact, the loss of Ca^2+^ is the key factor driving the decrease in total alkali content and surface basicity. This phenomenon may be attributed to the unique process control measures of Moutai-flavor Baijiu production, where acidic materials such as fermented grains and tail wine undergo acid–base neutralization during long-term production operations, leading to a gradual reduction in the total alkali content on the surface of the cooling yard (Zhu et al., 2024). To further elucidate the physical characteristics of the aged cooling yard surface, N_2_ adsorption–desorption analysis was performed. As shown in Supplementary Figure S7, the Type IV isotherm with an H3 hysteresis loop indicates the development of a meso/macroporous structure due to long-term physical wear. This increased surface roughness and porosity, characterized by a high volume adsorption at high relative pressure, likely provides a favorable physical habitat for microbial colonization, complementing the chemical selection pressure exerted by the decreasing alkalinity. The formation of a weakly acidic environment facilitates the inhibition of spoilage bacterial growth and promotes key enzymatic reactions (e.g., amylase and protease activities), thereby maintaining microecological balance and improving fermentation quality (Zhang et al., 2019; Wei et al., 2023).

Microbial community analysis revealed that the surface of the newly prepared TLCS cooling yard harbored higher ARA of bacteria such as *Aerococcus*, *Corynebacterium*_1, and *Alkalibacterium*, along with elevated levels of fungi including *Pichia*, *Aspergillus*, and *Cyberlindnera* (Figures 7c,d). Overall, the dominant fungal community structure showed minimal divergence from that of cooling yards under long-term production, further confirming the high resistance and tolerance of fungi to environmental stress (Zhang et al., 2022). In contrast, the composition of dominant bacteria differed significantly between new and aged cooling yards, with *Alkalibacterium* (16.18%) and *Nesterenkonia* (11.46%) dominating the newly prepared surface. Notably, *Nesterenkonia* was the primary signature taxon in the fermented grains of the 5-year-old group (Figure 2i). Previous studies have reported that *Alkalibacterium* is an obligate alkaliphile, unable to grow in low-pH environments but capable of thriving at pH > 11 (Nakajima et al., 2005). Similarly, *Nesterenkonia* exhibits alkaliphilic properties and thrives in alkaline conditions (An et al., 2025). In contrast, the ARA of *Lactobacillus* in the new cooling yard (2.17%) was substantially lower than that in the aged counterpart (35.59%). Therefore, the strongly alkaline surface environment of the new cooling yard may be a key factor contributing to the low ARA of *Lactobacillus* in pit-entry fermented grains. This phenomenon aligns with the physiological characteristics of *Lactobacillus*, which thrives in acidic to neutral environments. When the environmental pH is excessively high, it leads to the reversal of the transmembrane pH gradient, disrupts the proton motive force, and thereby inhibits its critical metabolic activities (Cotter and Hill, 2003).

## 4 Conclusion

This study employed high-throughput sequencing to characterize the microbial diversity in samples (Daqu, cooling yard, air, and fermented grains) collected from Moutai-flavor Baijiu production workshops with different operational ages. Results indicated that the ARA of *Lactobacillus* in fermented grains increased with workshop age, emerging as a key biomarker in the 30-year in-service facility. Source tracking analysis revealed a 74.60% community similarity between the cooling yard and fermented grains, along with an average contribution rate of 54.20%, suggesting that the cooling yard may act as the primary driver of the microbial community structure in fermented grains. Furthermore, comparative analysis of the physicochemical properties and microbiomes between new and aged cooling yards demonstrated that the high total alkali content on the surface of the new cooling yard promoted the enrichment of alkaliphilic bacteria (e.g., *Alkalibacterium* and *Nesterenkonia*) while inhibiting acidophilic *Lactobacillus*. Consequently, the ARA of *Lactobacillus* on the surface of the 30-year in-service cooling yard was 33.42% higher than that in the newly constructed counterpart. These findings confirm that the cooling yard microenvironment is a critical determinant of *Lactobacillus* abundance variations in pit-entry fermented grains across workshops of different ages. This insight provides a valuable theoretical foundation for regulating microbial community succession and assembly in newly commissioned workshops to improve base liquor quality.

## Data Availability

The raw sequence data reported in this paper have been deposited in the Genome Sequence Archive (Genomics, Proteomics & Bioinformatics 2025) in National Genomics Data Center (Nucleic Acids Res 2025), China National Center for Bioinformation / Beijing Institute of Genomics, Chinese Academy of Sciences. The ITS raw sequences are accessible under accession number CRA039282, and the 16S raw sequences are available under accession number CRA039275. Both datasets are publicly available at https://ngdc.cncb.ac.cn/gsa.

## Footnotes

1
http://huttenhower.sph.harvard.edu/galaxy/
