# Supplementary material for: Temporal heterogeneity of microbial ecosystems and its formation mechanisms in Moutai-flavor Baijiu fermentation
Source: Front Microbiol. 2026 Mar 12;17:1798174. doi: 10.3389/fmicb.2026.1798174 (PMC13017840; doi:10.3389/fmicb.2026.1798174)
Supplement: Supplementary file 1 [file Data_Sheet_1.docx]

Supplementary Material

## Supplementary Figures

**
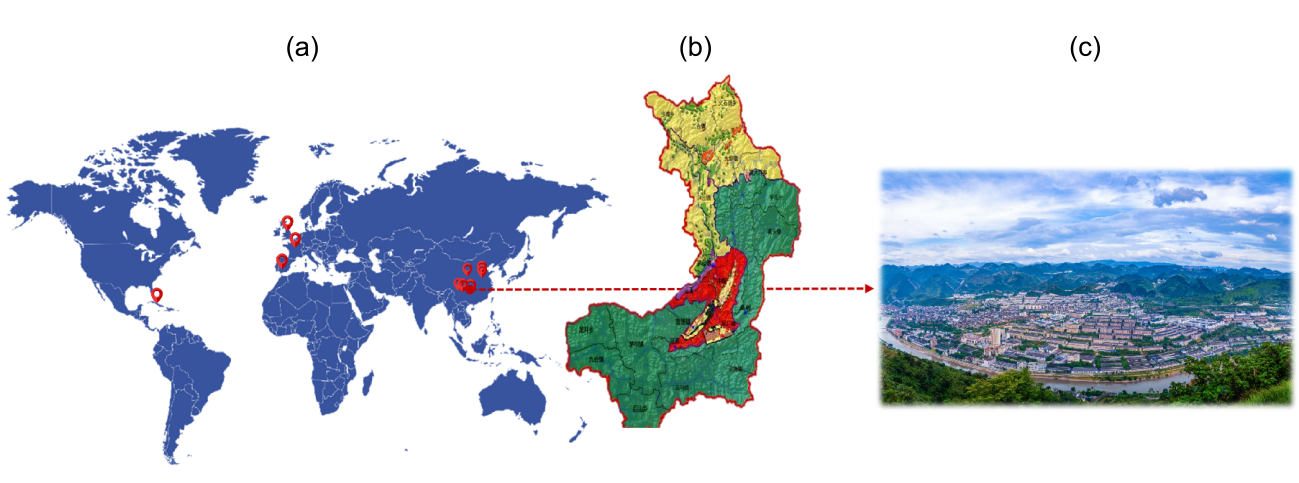
**

**Supplementary Figure 1.** Schematic Diagram of the Core Production Area of Moutai-Flavor Baijiu.

**
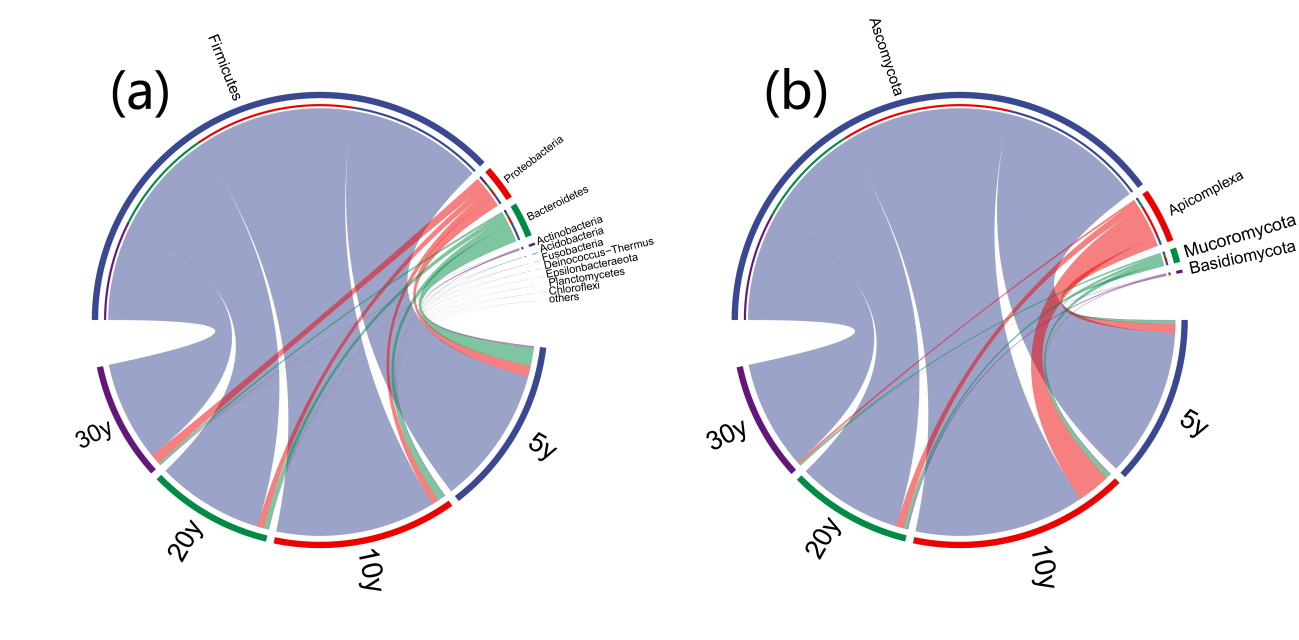
**

**Supplementary Figure 2.** Microbial Phylum-Level Analysis in Pit-Entry Fermented Grains: (a) Bacteria; (b) Fungi.

**
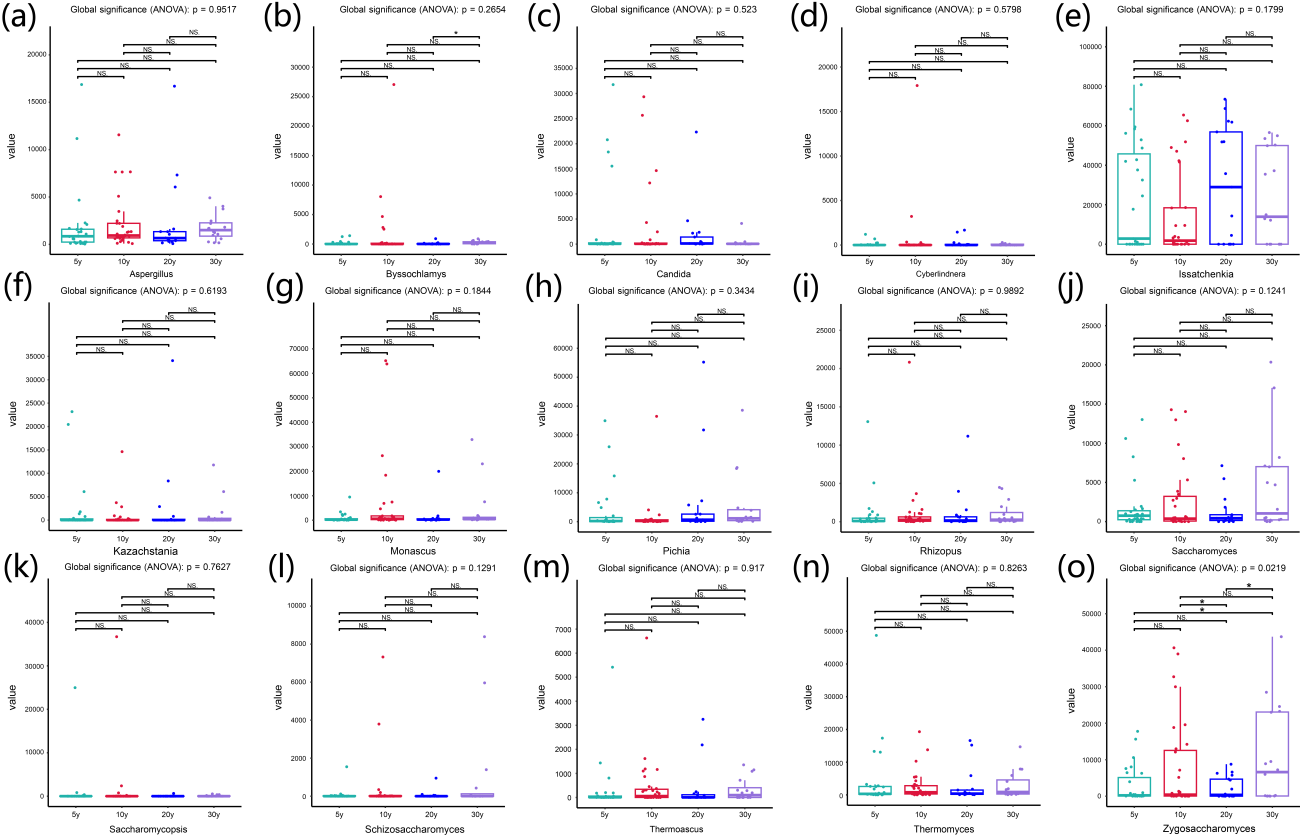
**

**Supplementary Figure 3.** Temporal Heterogeneity of Dominant Fungal Genera in Pit-Entry Fermented Grains.

**
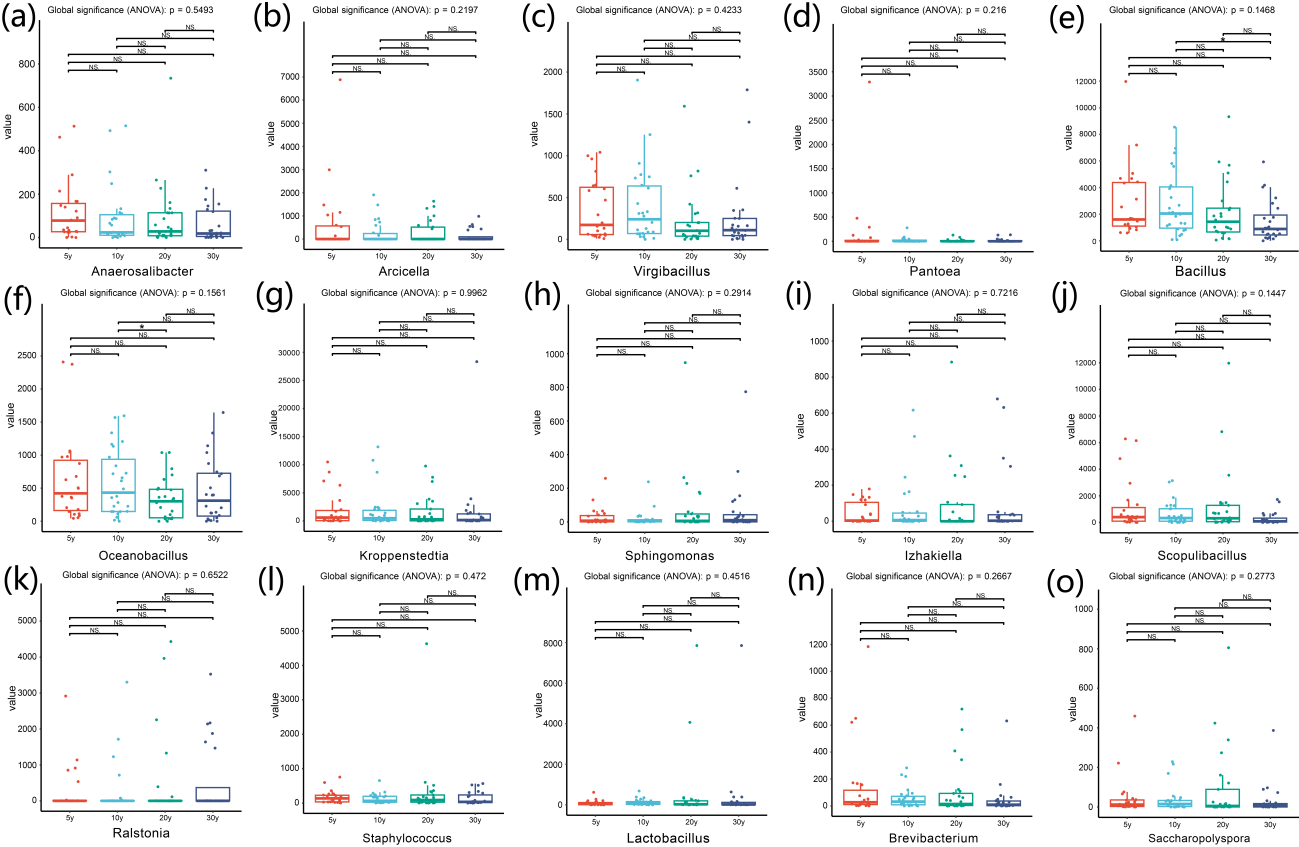
**

**Supplementary Figure 4.** Temporal Heterogeneity of Dominant Bacterial Genera in Daqu.

**
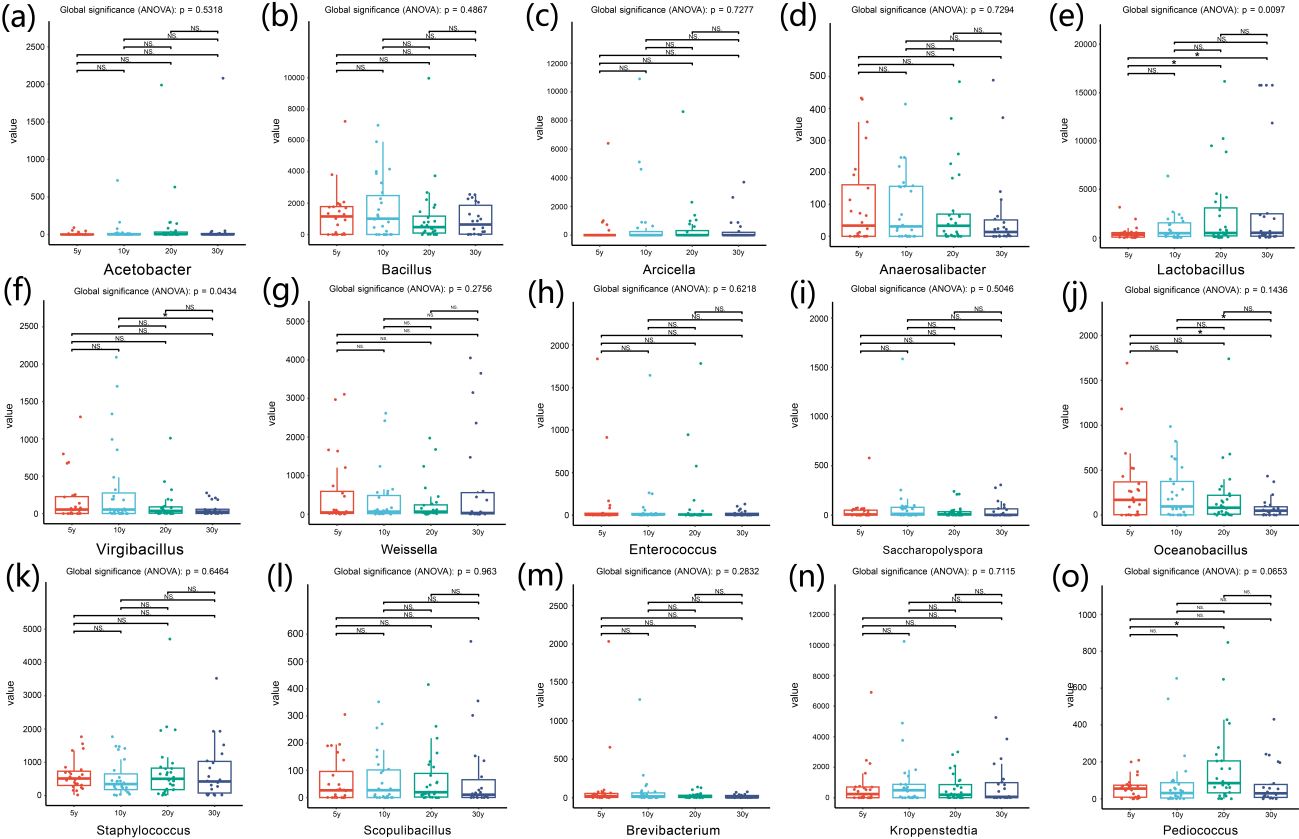
**

**Supplementary Figure 5.** Temporal Heterogeneity of Dominant Bacterial Genera in the Cooling Yard.

**
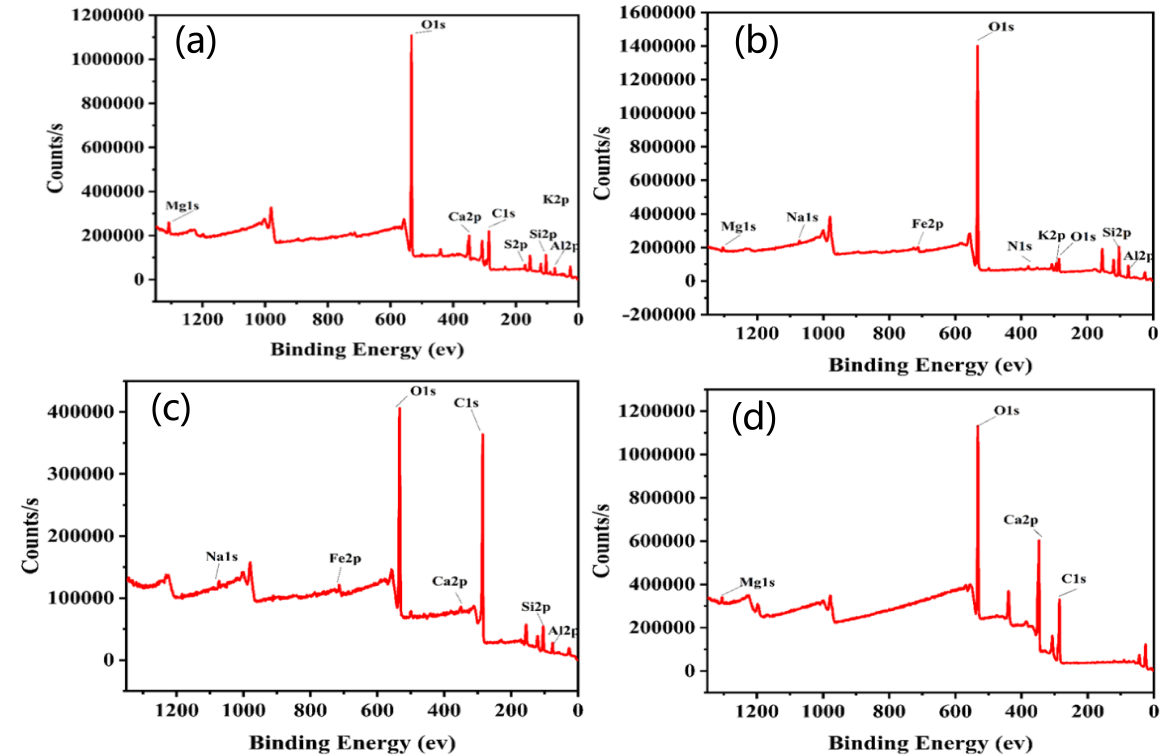
**

**Supplementary Figure 6.** X-ray Photoelectron Spectroscopy (XPS) of TLCS and its Components: (a)TLCS; (b) Purplish-red mud; (c) Cinder; (d) Quicklime.

**Table S1.** Temporal Heterogeneity of the Major Elemental Composition in the TLCS Cooling Yard.

| **Sample** | **Si (%)** | **Ca (%)** | **Al (%)** | **Si / Al** |
| --- | --- | --- | --- | --- |
| 5-year-old | 11.49 | 77.31 | 4.65 | 2.47 |
|  | 10.75 | 77.97 | 4.36 | 2.46 |
|  | 12.58 | 75.54 | 5.51 | 2.28 |
| 10-year-old | 9.97 | 67.74 | 4.99 | 2.03 |
|  | 14.53 | 69.92 | 6.15 | 2.36 |
|  | 14.43 | 65.26 | 6.84 | 2.12 |
| 20-year-old | 27.79 | 49.40 | 11.58 | 2.39 |
|  | 26.61 | 49.22 | 11.33 | 2.34 |
|  | 26.03 | 50.38 | 10.67 | 2.45 |
| 30-year-old | 41.62 | 29.47 | 17.09 | 2.43 |
|  | 33.78 | 38.10 | 13.63 | 2.47 |
|  | 27.74 | 32.81 | 13.56 | 2.04 |

**
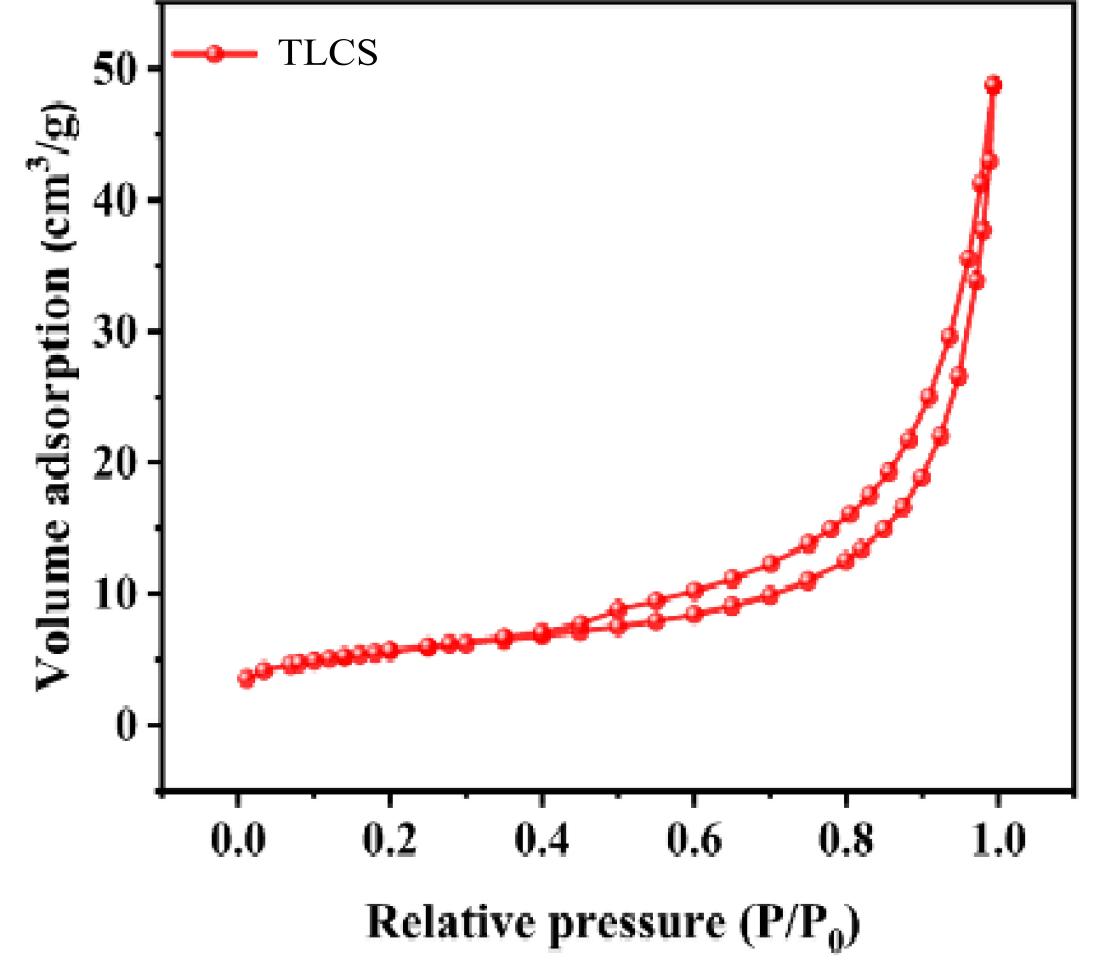
**

**Supplementary Figure 7.** N_2_ adsorption-desorption isotherms of the TLCS cooling yard.
